# Editorial: Recent advancement of the cardio-renal protective effects of SGLT2 inhibitors in people with and without diabetes

**DOI:** 10.3389/fmed.2023.1293372

**Published:** 2023-10-27

**Authors:** Satoru Kuriyama, Masaaki Nakayama

**Affiliations:** ^1^Jikei University School of Medicine, Tokyo, Japan; ^2^Division of Nephrology, St. Luke's International University, Tokyo, Japan

**Keywords:** SGLT2 inhibitor, type 2 diabetes, renal protection, chronic kidney disease, heart failure

Sodium glucose cotransporter 2 (SGLT2) inhibitors were originally developed for the treatment of type 2 diabetics and came into our clinical practice since 2014. At the beginning, we believed that the primary benefit of SGLT2 inhibitors could have resulted merely in a better blood glucose (BG) control in type 2 diabetics. However, apart from the metabolic effect, SGLT2 inhibitors provide substantial cardiovascular benefits which include reduction in heart failure (HF), hospitalizations for HF and death. These cardio-protection has been observed in both diabetics and non-diabetics. Moreover, SGLT2 inhibitors have apparent kidney protective effects that have been universally observed in people with and without diabetes. As a result of abundant clinical evidence, these drugs have recently positioned in a central place in guidelines of Diabetology and Nephrology worldwide. To date, lots of basic and clinical research works showed that the mode of actions is multifaceted with having still unclarified unknown mechanisms. To address this, it should be emphasized that many basic and clinical studies have currently been ongoing. In this Research Topic in Frontiers, we have published five papers including four reviews and a retrospective study which will enhance our knowledge and expand understanding on SGLT2 inhibitors. The followings are what we learn from our new reports.

Firstly, Zhu et al. explored the roles of SGLT2 inhibitors in acute heart failure (AHF) risk after acute myocardial infarction (AMI). The study was on a single-center, retrospective, and observational basis including 990 AMI patients comprising 386 non-ST-segment elevation myocardial infarction (NSTEMI) and 604 segment elevation myocardial infarction (STEMI) patients. The results showed that in NSTEMI patients, a lower proportion received SGLT2 inhibitors in the AHF group compared with the non-AHF group. Use of SGLT2 inhibitors was associated with reduced brain natriuretic peptide levels both in STEMI and in NSTEMI. In addition, SGLT2 inhibitors was associated with the reduced in-hospital AHF risk and has a strong protective effect against AHF in NSTEMI patients with hypertension. Furthermore, SGLT2i reduced the risk of in-hospital AHF for both diabetics and non-diabetics. These results suggest that SGLT2 inhibitors can reduce the risk of AHF in AMI patients. This novel study is suggestive of the SGLT2-mediated mechanisms in the pathogenesis of AMI.

Secondly, Cisneros-García et al. focused on non-traditional risk factors for end stage renal disease (ESRD) by reviewing 46 studies based on a scoping review methodology. In addition to the most remarkable risk factors for CKD, diabetes and hypertension, uncommon risk factors are also identified, which include dehydration, leptospirosis, heat stress, water quality etc., reported elsewhere. In this study, the non-traditional ESRD risk factors are newly depicted. Such factors include gender, ethnicity, erythematous systemic lupus (ESL), pesticide use and congenital and hereditary diseases in the urinary tract. The authors propose that it is necessary to put the issue on the table and add it to the public agenda in order to find multidisciplinary solutions. The study evokes a new interest in the field of CKD progression.

Thirdly, a review by Nishiyama and Kitada was especially intriguing. They have previously shown that SGLT2 inhibitors induce antihypertensive effects with decreased sympathetic nerve activity associated with transient natriuresis. They also found treatment with an SGLT2 inhibitor improves renal ischemia by producing vascular endothelial growth factor-α in the renal tubules. Others have suggested that ketone body production, changes in glomerular hemodynamics, and intrarenal metabolic changes and a reduction in oxidative stress due to decreased tubulointerstitial glucose levels may also be involved in the renoprotective effects of SGLT2 inhibitors. In their review, they summarize the mechanism responsible for the SGLT2 inhibitor-induced renoprotective effects, including their novel hypothesis regarding an “aestivation-like response”, which is a biological defense response to starvation. This concept is anew and deserves to be discussed.

Fourthly, Zhang et al. made a review on the etiology of immunoglobulin A nephropathy (IgAN), the most common primary glomerulonephritis and the leading cause of ESRD. The current widely accepted framework for its pathogenesis is the “multi-hit hypothesis”. In this interesting review, they discussed the intrarenal inflammation in IgAN by focusing on four main types of cells including mesangial cells, endothelial cells, podocytes, and tubular epithelial cells (TECs). Their interactions are complex. Namely, activation of mesangial cells by galactose deficient-IgA1 (Gd-IgA1) deposition with enhanced cellular proliferation, extracellular matrix (ECM) expansion and inflammatory response plays a central role in the pathogenesis of IgAN. Regional immune complexes deposition and mesangial-endothelial crosstalk result in hyperpermeability of endothelium with loss of endothelial cells and infiltration barrier proteins, and recruitment of inflammatory cells. Podocyte damage is mainly derived from mesangial-podocytic crosstalk, in which tumor necrosis factor-α (TNF-α), transforming growth factor-β (TGF-β), renin-angiotensin-aldosterone system (RAAS) and micro-RNAs are the major players in podocyte apoptosis and disorganization of slit diaphragm (SD) related to proteinuria in IgAN patients. In addition to filtrated proteins into tubule-interstitium and mesangial-tubular crosstalk involved in the injury of TECs, retinoic acid has been discovered innovatively participating in TEC injury. In a clinical viewpoint, dapagliflozin provides substantial evidence of renal protection in patients with IgAN (DAPA-CKD) (1), therefore the possibility that SGLT2 inhibitors act on the kidney to slow down the progression via the above-mentioned immunological mechanisms is of particular interest and is worth evaluating in the future projects.

Finally, Tornyos et al. performed a network meta-analysis on gliflozins. The primary endpoint of interest was the rate of HF-related hospitalization (HHF) and the composite of HHF with CV mortality (HHF + CVD). Secondary outcomes included major adverse cardiac events (MACE), CV- and overall mortality, myocardial infarction (MI), and stroke. Twenty-nine studies randomizing 88,418 patients were identified. Gliflozins reduced the risk of HHF [RR: 0.72 (0.69; 0.76)] and HHF + CVD [RR: 0.78 (0.75; 0.82)]. The risk of MACE and its component also improved significantly except for stroke. The network analyses did not explore major differences among the individual substances. The only exception was sotagliflozin which appeared to be more effective regarding HHF + CVD, stroke, and MI compared to ertugliflozin, in HHF + CVD and stroke compared to dapagliflozin, and in stroke endpoint compared to empagliflozin. They concluded that a group effect of gliflozins beneficial in a wide spectrum of patients with a risk of HF development. In addition, the risk of major adverse events is also reduced with SGLT2 inhibition.

All in all, we believe that these papers will contribute further to understand the recent advancement of the cardio-renal protective effects of SGLT2 inhibitors.

## Author contributions

SK: Writing—original draft. MN: Writing—review and editing.

## References

[1] Wheeler DC Toto RD Stefánsson BV Jongs N Chertow GM Greene T. A pre-specified analysis of the DAPA-CKD trial demonstrates the effects of dapagliflozin on major adverse kidney events in patients with IgA nephropathy. Kidney Int. (2021) 100:215–24. 10.1016/j.kint.2021.03.033 33878338

